# Career Planning in Public Health

**DOI:** 10.3389/fpubh.2014.00250

**Published:** 2014-12-19

**Authors:** Joel M. Lee

**Affiliations:** ^1^John A. Drew Professor of Healthcare Administration, The University of Georgia College of Public Health, Athens, GA, USA

**Keywords:** public health, career choice, book reviews as topic, guidelines as topic, future

In contrast to many other professions, prospective public heath students and particularly undergraduate students are frequently uninformed about the career opportunities available to them. *101 Careers in Public Health* seeks to address this deficiency.

*101 Careers in Public Health* is one of the series of Springer Publishing’s 101 Careers books that includes guides for Healthcare Management, Gerontology, Psychology, Counseling, and Social Work. The book does provide information on 101 public health career paths, but offers additional information for the novice briefly defining what public health is, educational pathways including baccalaureate, masters, doctoral, and combined degrees, and finding jobs in public health.

Following the introductory chapters, the book is divided into 19 career chapters addressing opportunities in infectious disease, chronic disease and cancer, public safety, maternal and child health, pharmaceutical and drug safety, environmental health and water safety, occupational health and safety, food safety and nutrition, disaster preparedness and response, health communication, education, public mental health, public health law, regulations, and policies, non-profit organizations, public health administration and leadership, global health, and a category called “off the beaten path.” Each chapter offers four to eight individual job descriptions. For each job, there is a template of a position description, education and certification requirements, core competencies, compensation, workplaces, an employment outlook, and citation of sources for further information. Thirty of the careers are followed by a profile of a practitioner based upon an interview that includes responses to a common set of questions about the job: a typical day, education, the path to the current job, advice, future plans, the worst or most challenging part of the job, and advice for those interested in the job.

The book does indeed address 101 careers in public health, including the traditional public health careers such as epidemiologist, health educator, and biostatistician, and appropriate entry positions for baccalaureate graduates (health educator, specialist in poison information, community health worker, and health teacher). Additional opportunities focus on dual degree positions such as public health veterinarians, dentists, nurses, pharmacists, lawyers, environmental engineers, social workers, and occupational medicine physicians. Others focus on careers more appropriate to master’s level education in public health for practice (injury prevention specialist, deputy director/family health services, hydrologist, emergency preparedness specialist) or doctoral level education in public health (behavioral scientist, professor, or researchers in disaster preparedness, bioterrorism, mental health, and outcomes).

Other opportunities are more peripheral to public health, in careers such as journalism, marketing, or as a health teacher. Additional group is aspirational positions in public health including dean of a school of public health, federal agency director, Surgeon General of the United States, NGO founder/director, and professional association director. The “off the beaten path” category includes positions such as dance instructor, urban planner, hospital administrator, and chef. The book concludes with a brief discussion of the public health future and career opportunities related to health reform, climate change, changing populations, and new media.

While the introductory and concluding sessions provide a brief overview of what public health is, education for public health careers, finding jobs, and the future, each of these sections is short and may best serve as an introductory first step rather than a detailed guide. To achieve the target of 101 careers, the range of opportunities may be a bit stretched, and a large number of the jobs require clinical credentials in medicine or another health profession. Opportunities for graduates with a master of public health degree as their entry credential is the most modest group of career choices offered.

Only two other books on public health careers have been published. *Advancing Healthy Populations: The Pfizer Guide to Careers in Public Health* (1) follows a similar approach of profiles of 25 public health professionals in a less structured narrative format without the individual position descriptions. The book also includes five “Thought Pieces” by senior public health practitioners. Originally distributed at no charge, the book no longer appears to be in print, but is available online at several sites. *Public Health: Career Choices That Make a Difference* (2), uses a similar approach to *101 Careers* offering chapters addressing public health and occupations, the future, and appendices of resources, but organizes its career information around more general chapters addressing categories of career paths in administration, environment and occupational health, and epidemiology and disease control. Consequently, *101 Careers* is the most recent of these books, accessible to students, and offers a blend of these two approaches for the novice.

Each of these guides will offer value and insight to those individuals with a curiosity about public health careers and the educational requirements leading to positions. Despite minor limitations, *101 Careers in Public Health* will serve as an excellent introduction to opportunities for undergraduates exploring public health, and may assist a high school or undergraduate student develop an appreciation for baccalaureate majors and minors, or graduate education. Student advisors in high schools and colleges will find the book useful as a tool to acquaint students with pathways to a career in public health when used in conjunction with web sites such as http://www.aspph.org/discover/, www.ceph.org, and www.cdcfoundation.org/content/what-public-health. Faculty who are shaping undergraduate public health programs will find the range of careers presented useful as a basis to help students understand public health professional competencies and knowledge needed for entry to the field.

## Conflict of Interest Statement

The author declares that the research was conducted in the absence of any commercial or financial relationships that could be construed as a potential conflict of interest.
